# Reconstruction of real and simulated phylogenies based on quartet plurality inference

**DOI:** 10.1186/s12864-018-4921-5

**Published:** 2018-08-13

**Authors:** Eliran Avni, Sagi Snir

**Affiliations:** Department of Evolutionary Biology, University of Haifa, 199 Aba Khoushy Ave. Mount Carmel, Haifa, 3498838 Israel

**Keywords:** Phylogenetic reconstruction, Quartet plurality, Horizontal gene transfer, Supertree reconstruction, Prokaryotic evolution

## Abstract

**Background:**

Deciphering the history of life on Earth has long been regarded as one of the most central tasks in biology. In past years, widespread discordance between the evolutionary histories of different groups of orthologous genes of prokaryotes have been revealed, primarily due to horizontal gene transfers (HGTs). Nonetheless, evidence that support a strong tree-like signal of evolution have been uncovered, despite the presence of HGT events. Therefore, a challenging task is to distill this tree-like signal from the noise induced by all sources of non-tree-like events.

**Results:**

In this work we tackle this question, using real and simulated data. We first tighten a recent related theoretical result in this field. In a simulation study, we infer individual quartet topologies, and then use the inferred quartets to reconstruct simulated species trees. We demonstrate that accurate tree reconstruction is feasible despite surprisingly high rates of HGT. In a real data study, we construct phylogenies of two sets of prokaryotes, and show that our tree reconstruction scheme is comparable with (and complementary better than) other commonly used methods.

**Conclusions:**

Using a blend of theoretical and empirical investigations, our study proves the feasibility of accurate quartet-based phylogenetic reconstruction, the vast impact of HGT events notwithstanding.

**Electronic supplementary material:**

The online version of this article (10.1186/s12864-018-4921-5) contains supplementary material, which is available to authorized users.

## Background

The reconstruction of trees of ancestor-descendant relationships, also known as gene trees, is an important step in many phylogenomic analyses. When gene trees for each family of orthologous genes in a dataset were reconstructed, analyses have revealed widespread discordance between those trees [1]. Various factors may lead to incongruences between the inferred gene histories. Some are technical in nature, such as statistical errors in gene tree estimation, and others stem from biological processes, for example duplications and losses in gene families, hybridization events, incomplete lineage sorting, and horizontal genetic transfers [2–4], the latter being a major driving force of evolution in the prokaryotic world. Hence, the advent of High-throughput sequencing and the exponentially accumulating genetic data, once thought to help us decipher the history of life on Earth, now only seem to show that unravelling this history is a harder task than initially thought.

In this work we focus on horizontal gene transfer (HGT), that is, the transfer of genes not between parent and offspring through standard vertical transmission, but between two contemporaneous organisms. HGT is largely mediated by a variety of mobile elements such as viruses (bacteriophages), plasmids, and transposons. Besides being especially prevalent in prokaryotic evolution, HGT also influences the study and research of infectious diseases [5], as it plays an important role in microbial adaptation to antibiotics. Estimates of the fraction of genes that have undergone HGT are heatedly debated, and some are as high as 99% [1, 6]. Indeed, a number of researchers now doubt the very meaningfulness of the Tree of Life concept [7–13]. However, despite the fact that HGT tangles the traditional universal Tree of Life and turns it into a network of relationships, many believe that an underlying species tree can still be reconstructed, and there is ample evidence to support that claim [1, 14–20].

Typically, the underlying species phylogeny of prokaryotes is inferred by constructing gene trees for genes that are regarded as resistant to HGT, often ribosomal RNA genes. However, even such genes are subjected to HGT, obfuscating the tree-like trend of evolutionary relationships [21–24]. Unfortunately, since these genes are highly conserved, the amount of evolutionary signal they provide is frequently insufficient for a reliable classification within a genus or even a family. Therefore, it was suggested to construct the underlying species phylogeny by considering a multitude of carefully constructed and highly accurate gene trees (usually of smaller size) and subsequently amalgamating them together, an approach denoted as the *supertree* operation [25, 26]. In most cases, the supertree operation is a computationally intractable problem (NP-hard or NP-complete), which necessitates the usage of heuristic approaches.

As evolution is time driven, phylogenies are naturally rooted, reflecting ancestor-descendant relationships. In a rooted tree, the basic unit of information is a rooted triplet: a tree over three species, where two species are sister leaves and the third forms an outgroup. However, most phylogenetic methods construct an unrooted tree where the phylogenetic information represents splits rather than ancestry, since raw data, i.e. sequences of DNA or amino acids, does not convey information about the timescale. In such an unrooted setting, the basic unit of information is an unrooted quartet tree or simply a quartet. Hence, one of the simplest cases of the supertree problem in an unrooted setting is when the input is comprised solely of quartets and the goal is to amalgamate these quartets into a single tree. This task, also known as *quartet-based supertree* or *quartet amalgamation*, has attracted interest from practitioners and is relevant to many tasks in phylogenetics [27–30]. The inference of such quartets is usually done accurately and rigorously from raw data [31–35]. Due to its fundamental role, quartet amalgamation has been the focus of theoretical studies as well [36–38]. It is noteworthy that a given set of quartets is not necessarily *compatible* (or *consistent*) in the sense that it does not necessarily agree with some single tree. Finding the largest compatible subset of a given quartet is computationally intractable (NP-complete, [39]), and the best-known solution to the general problem remains a random tree, satisfying (only) one third of the input, intensive efforts notwithstanding [40, 41].

In [42], Roch and Snir tackled the problem of reconstructing the phylogeny of a single quartet. Using the plurality inference rule (see details in “Preliminaries” section), and assuming that HGT events are consistent with a certain Poisson process of a constant rate, they showed that such a reconstruction can be achieved with high probability, even when the number of HGT events per gene is surprisingly high ($O\big (\frac {n}{\text {log}{n}}\big)$, where *n* is the number of species). Since the number of edges in a gene tree is *O*(*n*), this means that the number of HGT events can be almost proportional to the number of edges without destroying the overall tree signal.

Here we extend the *plurality inference rule* into a complete tree reconstruction scheme, instead of using it solely as a quartet oracle. We evaluate the quality of the reconstructed tree, using either simulated or real biological data. We first recollect the bound of [42] and use it to evaluate the probability of simultaneous accurate inference of a large number of quartets induced by a given collection of species trees, that in turn yields perfect tree reconstruction. Next, we show via detailed simulations, that the plurality inference rule leads to accurate tree reconstruction even when the number of HGT events affecting species’ evolution is much larger than what the theory predicts. In the last part of the paper, we show that the plurality inference rule can be turned into a viable tool for phylogenetic reconstruction of real data. We do this by applying the principles of the theoretical and simulative work to two sets of prokaryotes: one consists of 100 archaea and bacteria, representing prokaryotic life as a whole, and the other consists of 97 bacteria, that share a unique property - each of the 97 bacteria has at least one gene toxic to *E. coli*. Quartet plurality inference was already used as a basis for real data tree reconstruction in the past [43], but our usage is novel due to the magnitude of the species sets involved, and the diverse patterns of HGT exhibited by the different types of genes (toxic versus non-toxic).

## Methods

### Preliminaries

Here we present the relevant and necessary background information of our work.

**Phylogenetic trees** - For a set of species (or *taxa*) $\mathcal {X}$, a phylogenetic $\mathcal {X}$-tree *t* is a tree for which there is a one to one correspondence between $\mathcal {X}$ and the set of leaves of *t* - $\mathcal {L}(t)$. The removal of an edge from a tree disconnects the tree into two subtrees and induces a *split* on the taxa set. The split $(U,\mathcal {X}\setminus U)$ identified by the edge *e* is denoted as *e*_*U*_ or $e_{\mathcal {X}\setminus U}$ alternatively. Let *t* be an $\mathcal {X}$-tree and $\mathcal {A}\subseteq \mathcal {X}$ be a subset of $\mathcal {X}$. We denote by $t|_{\mathcal {A}}$ the subtree that is *induced* by $\mathcal {A}$ on *t* and is thus obtained: First, all the leaves in $\mathcal {X}\setminus \mathcal {A}$, as well as paths leading exclusively to them, are removed. Next all internal nodes (necessarily in paths connecting leaves from $\mathcal {A}$) with degree two are contracted.

**Consistent trees and supertrees** - For two trees *T* and *t*, we say that *Tsatisfiest* (alternatively, *t* is induced by *T*) if $\mathcal {L}(t)\subseteq \mathcal {L}(T)$ and $T|_{\mathcal {L}(t)}=t$, otherwise, *t* is *violated* by *T*. For a set of trees $\mathcal {T}=\{t_{1},\ldots,t_{k}\}$ with possibly overlapping leaves, we say that $\mathcal {T}$ is *consistent* if there exists a tree *t*^∗^ that satisfies every tree $t_{i}\in \mathcal {T}$. Otherwise, $\mathcal {T}$ is *inconsistent*. The problem of finding such a consistent tree *t*^∗^ is known as the *supertree problem*. It is generally NP-complete [39].

Tree amalgamation, i.e. combining several trees into a unified supertree, may be done in several ways (see [25] for some examples). In general, each such procedure, also known as a *supertree method*, attempts to find a tree that maximizes a given function relating to the input. Except in a few special cases, this operation is computationally intensive (NP-hard), and heuristic approaches must be employed.

**Quartets and the*Maximum Quartet Consistency*problem** - A Tree *t* is *rooted* if all edges are directed away from a given node, the *root*. When edges are undirected and there are no ancestor-descendant relationships between the nodes, the tree is *unrooted*. In this work we deal only with unrooted trees. In this context, the basic unit of information is a tree with four taxa, a *quartet* tree. A quartet tree with taxa {*a*,*b*,*c*,*d*} is denoted by *a*,*b*|*c*,*d* if a split ({*a*,*b*},{*c*,*d*}) is induced by one of the tree’s edges. More generally, a quartet *q*=*a*,*b*|*c*,*d* is satisfied by a tree *t* if *t* has a split separating *a*,*b* from *c*,*d*. Notice that a rooted triplet is always transformed to a star tree in the unrooted case, which contains no biological information. For the same reason, unresolved quartets, i.e. quartets whose topology is a star, are ignored in this paper. A common special case of the supertree problem is when the input consists solely of quartets and the objective is to find a tree that satisfies the maximum number of quartets. This is denoted as the *Maximum Quartet Consistency* (MQC) problem.

In this work we chose to use the weighted Quartet MaxCut (wQMC) heuristic (see [44]) as our supertree method. wQMC, a weighted extension of QMC [45, 46]), receives as input a collection of weighted quartet trees and aims at finding a tree that maximizes the total weight of the input quartets it satisfies. In “Reconstructing the species tree based on gene trees” section we elaborate more on this issue.

**Characters and perfect trees** - Given a set of leaves $\mathcal {L}$, a *character* on $\mathcal {L}$ is a partition of $\mathcal {L}$, i.e., a division of $\mathcal {L}$ into disjoint subsets. Each of the subsets in a given character is called *a state*. We say that a tree *t* with leaves $\mathcal {L}$ is *perfect* with respect to a character *c* (equivalently, *tdisplaysc*) if for every two states *r*_1_ and *r*_2_ in *c*, the nodes having states *r*_1_ and *r*_2_ form two disjoint subtrees of *t*. Informally, a perfect tree is a tree that induces a perfect separation between the states of the character in question. A set of characters is called *compatible* if there exists a tree which displays them all simultaneously. Determining if a given collection of characters is compatible or not is called the *perfect phylogeny problem* (or *character compatibility problem*). It is, in general, NP-complete [39].

**The plurality inference rule** - The plurality inference rule functions as a quartet oracle that helps to reconcile conflicting gene trees. As mentioned earlier, gene trees often exhibit remarkable discordance when different gene clusters are studied. Thus, while a given gene tree may induce one topology per one 4-taxa, say *a*,*b*|*c*,*d*, *a*,*c*|*b*,*d* or *a*,*d*|*b*,*c* for the set {*a*,*b*,*c*,*d*}, when examining a collection of gene trees, different genes may induce different quartet topologies. In a study that involves a collection of gene trees, the number of trees satisfying each topology is counted. The plurality inference rule assumes that the most prevalent topology is identical to the original topology in the species tree (an assumption that is justified by [42]). That topology is denoted the “plurality topology” and kept for further analysis.

**Quartet fit** - The Quartet fit tree similarity measure ([47], abbreviated to Qfit in this paper) is a measure that receives two trees (with identical leaves) and returns the percentage of quartets shared by them. More precisely, for two trees *t*_1_,*t*_2_ we define

1 $$ \text{Qfit}\left(t_{1},t_{2}\right)=\frac{g}{g+b}  $$

where *g* is the number of 4-taxa sets for which the quartet topologies induced by the two trees are the same, and *b* is the number of 4-taxa sets for which the quartet topologies induced by the two trees are different. Unresolved quartets (that is, quartets for which the induced topology is a star topology) are ignored. This enables one to differentiate between unresolved quartets and quartets whose induced topologies by the two trees are different. One can apply (1) to find the Qfit similarity score between a set of quartets and a tree in an obvious way.

**Robinson-Foulds symmetric difference** - The Robinson-Foulds symmetric difference ([48], abbreviated to RF in this paper) measures the distance between two trees. Assuming that the two trees have the same leaves set, we define:

2 $$ \text{RF-distance}\left(t_{1},t_{2}\right)=\frac{b_{1}+b_{2}}{S_{1}+S_{2}}  $$

where *S*_1_ is the number of non-trivial splits in *t*_1_, and *b*_1_ is the number of *t*_1_ tree splits that are not induced by any one of the edges of *t*_2_. Similarly, *S*_2_ and *b*_2_ are defined in an obvious way. In order to use RF as a measure of similarity instead of difference, the number of non-trivial splits shared by the two trees in question was counted. Thus, RF was defined in this paper as

3 $$ \text{RF}\left(t_{1},t_{2}\right)=1-\frac{b_{1}+b_{2}}{S_{1}+S_{2}}.  $$

RF was implemented in Phylip [49].

### The simulation procedure

Ten random model trees over *n* taxa (for *n* = 10, 20,…100) were produced based on the Yule model [50]. Accordingly, each edge in the species trees was assigned a length. As the created model trees constitute an analogue to evolutionary species trees, they are referred to as species trees in “Simulation results” section, where it is clear from the context that computer generated species trees are dealt with. Subsequently, “gene” trees were generated based on the species trees. As in [42], each gene tree was created when a species tree was subjected to an HGT process, which is, a series of HGT events, consistent with a Poisson process of a constant rate. Specifically, we assume that the number of recipients of HGTs per one unit length is a Poisson distributed random variable with parameter *λ* defined by the user. Once a recipient of HGT was chosen, the donor of HGT was selected randomly and uniformly from all the contemporaneous species of the recipient, and then an HGT event was simulated by a Subtree Pruning and Regrafting (SPR) operation. For each simulated species tree we generated ten families of gene trees, based on ten different rates of HGT (*λ*=0.1, 0.2,…,1.0), each family consists of 2500 gene trees. For more details, see Additional file 1.

### Reconstructing gene trees based on protein sequences

The reconstruction of a phylogeny from a given collection of protein sequences is a two-staged process: 1) aligning the sequences; and 2) constructing the phylogeny based on the results of the alignment. The alignments were carried out using muscle [51]. Due to the large number of genes in the study (close to 3600), the construction of gene trees was carried out using fasttree [52] (using the Whelan-Goldman model of amino acid evolution [53]). The exception to that rule was the 16s-based phylogeny, for which an alignment file was downloaded from the RDP website [54] and a tree was subsequently constructed using RAxML (version 7.0.4 [55], assuming the GTR- *Γ* model [56]).

### Reconstructing the species tree based on gene trees

As mentioned above, wQMC was used in order to reconstruct species trees based on gene trees, in both simulated and real data settings. Since wQMC is a quartet-based reconstruction algorithm, it was necessary to transform the given gene trees into a suitable input for wQMC. Therefore, when examining a set of four leaves (4-taxa), its quartet topology was inferred based on the *plurality inference rule* defined above, and the resulting plurality topology was added to the input of wQMC.

## Results

Here we present the results of our study. Motivated by the work of [42], we start by further investigating the theoretical aspects of the quartet plurality inference rule mentioned above. Next, we examine the effects of HGT events on computer generated species trees and gene trees. We show that the number of quartet topologies that are inferred accurately using the plurality rule is substantially larger than the number of quartets shared by any two specific species tree and gene tree. This effect of the plurality rule on the quality of quartet inference can be dramatic even when a relatively small number of gene trees (several dozens) is considered in the analysis. Moreover, using wQMC as a supertree method, we demonstrate that accurate species tree reconstruction is feasible when the frequency of HGT events is much higher than predicted in [42]. In the last part of this section, we use the plurality rule to construct phylogenies of two sets of prokaryotes. We show that the phylogenies constructed using the plurality rule achieve similar scores (and sometimes better scores) compared to other hypothesized phylogenies in a number of tests.

### Using the plurality inference rule to reconstruct complete species trees

As mentioned earlier, the plurality inference rule is supported by a theoretical result: For a given species tree, gene tree, and 4-taxa, let us define “success” as the event in which the species tree and the gene tree induce the same topology on a that 4-taxa. In [42], it was proven that for any species tree, any gene tree, and any 4-taxa,

4 $$ Pr\left({~}^{\prime\prime}\text{success}^{\prime\prime}\right) \ge e^{-\lambda l}  $$

where *l* is the total length of the subtree induced by the 4-taxa and *λ* is the HGT rate (see “The simulation procedure” section). The authors base their result on the following assumptions:

1. The number of recipients of HGT per one unit length in the species tree is a Poisson distributed random variable with parameter *λ*.
2. For each recipient of HGT, the donor of HGT is selected randomly and uniformly from all the species co-existing with the recipient.

In order for the majority of gene trees to induce the correct quartet topology, let us require the probability of “success” to be at least 50%, and denote the height of the tree (i.e. the time past between the root and the leaves) as *h*. In this case, clearly *l*≤4*h*, hence *e*^−*λl*^≥*e*^−4*λh*^, and our requirement is met whenever *e*^−4*λh*^>0.5, or

5 $$ \lambda<\frac{\text{ln}2}{4h}\,.  $$

This implies that if (5) holds, then for every 4-taxa, the quartet topology induced by the species tree has at least a 50% chance of being induced by any given gene tree. Hence, if (5) is satisfied then the plurality rule enables one to infer all quartets correctly (with high probability), and then reconstruct the species tree correctly, provided that a large enough collection of gene trees is available for analysis. The following lemma summarizes this discussion.

#### **Theorem 1**

Under assumptions 1 and 2 above, for every tree *t* with *n* leaves and height *h*, and every *λ* that satisfies (5), the probability of inferring all quartet topologies correctly is at least $1-{n \choose 4}\text {exp}\left (-2\left (e^{-4\lambda h}-\frac {1}{2}\right)^{2}m\right)$, where *m* in the number of gene trees in the analysis.

#### *Proof*

Let us assume we have a species tree *t* and a collection of *m* gene trees, denoted *g*_1_, *g*_2_,…,*g*_*m*_. We fix a 4-taxa {*a*,*b*,*c*,*d*} and, for *k*=1,…,*m*, define

$${{}\begin{aligned} X_{k}\,=\,\left\{ \begin{array}{rcl} 1 & & g_{k}\text{ and }t\text{ induce the same quartet topology on }\{a,b,c,d\}\\ 0 & & \text{otherwise.} \end{array}\right. \end{aligned}} $$

Clearly, the *X*_*k*_-s are independent and identically distributed random variables (IIDs). We recall that according to Heoffding’s inequality [57], for every *ε*>0 and every collection of 0/1 IIDs we have

6 $$ Pr \left(\frac{1}{m}\sum\limits_{k=1}^{m}X_{k}-\frac{1}{m} \mathbb{E}\sum\limits_{k=1}^{m}X_{k}<-\varepsilon \right) \le\text{exp}\left(-2m\varepsilon^{2}\right).  $$

Setting *δ*=*mε*, the following holds for every *δ*>0:

7 $$ {{} \begin{aligned} Pr \left(\sum\limits_{k=1}^{m}X_{k}-\mathbb{E}\sum\limits_{k=1}^{m}X_{k}<-\delta \right) & = Pr \left(\sum\limits_{k=1}^{m}X_{k}-\mathbb{E}\sum\limits_{k=1}^{m}X_{k}<-m\varepsilon \right) \\ & = Pr \left(\frac{1}{m}\sum\limits_{k=1}^{m}X_{k}\,-\,\frac{1}{m}\mathbb{E}\sum\limits_{k=1}^{m}X_{k}\!<-\!\varepsilon \right) \\ & \le \text{exp}\left(-2m\varepsilon^{2}\right)  \\ & = \text{exp}\left(\frac{-2m\delta^{2}}{m^{2}}\right) \\ & = \text{exp}\left(\frac{-2\delta^{2}}{m}\right). \end{aligned}}  $$

We denote the expectation of *X*_*k*_ as *μ*. We may write

8 $$ {{} \begin{aligned} Pr \left(\sum\limits_{k=1}^{m}X_{k}<\frac{m}{2} \right) & = Pr \left(\sum\limits_{k=1}^{m}X_{k}-m\mu<\frac{m}{2}-m\mu \right)\\ & = Pr \left(\sum\limits_{k=1}^{m}X_{k}\,-\,\mathbb{E}\sum\limits_{k=1}^{m}X_{k}<-\left(\mu-\frac{1}{2} \right) m \right). \end{aligned}}  $$

We set $\delta =\left (\mu -\frac {1}{2}\right)m$ and recall that *μ*=*Pr*(“success”)≥*e*^−*λl*^≥*e*^−4*λh*^. Since we assume (5) holds, *e*^−4*λh*^>0.5 is valid as well, hence *μ*≥*e*^−4*λh*^>0.5 and *δ* is positive. Combining (7) and (8), we get

9 $$ {\begin{aligned} Pr \left(\sum\limits_{k=1}^{m}X_{k}<\frac{m}{2} \right) & = Pr \left(\sum\limits_{k=1}^{m}X_{k}-\mathbb{E}\sum\limits_{k=1}^{m}X_{k}<-\left(\mu-\frac{1}{2} \right) m \right)\\ & = Pr \left(\sum\limits_{k=1}^{m}X_{k}-\mathbb{E}\sum\limits_{k=1}^{m}X_{k}<-\delta \right)\\ & \le \text{exp}\left(\frac{-2 \delta^{2}}{m}\right)\\ & = \text{exp}\left(\frac{-2 \left(\mu-\frac{1}{2} \right)^{2}m^{2}}{m}\right)\\ & = \text{exp}\left(-2 \left(\mu-\frac{1}{2} \right)^{2}m\right) \\ & \le \text{exp}\left(-2 \left(e^{-4\lambda h}-\frac{1}{2} \right)^{2}m\right) \end{aligned}}  $$

which gives an upper bound on the probability of inferring a quartet’s topology *incorrectly*, since if ${\sum \nolimits }_{k=1}^{m}X_{k} \ge \frac {m}{2}$, the correct topology is inferred. If the number of leaves in *t* is *n* then there are $n \choose 4$ possible quartets and the probability of inferring at least one quartet topology incorrectly is bounded from above by ${n \choose 4}\text {exp}\left (-2\left (e^{-4\lambda h}-\frac {1}{2}\right)^{2}m\right)$, hence we deduce that

10 $$ {{} \begin{aligned} Pr(\text{all quartets are inferred correctly}) &\ge 1-{n \choose 4}\text{exp}\\&\left(\,-\,2\left(e^{-4\lambda h}-\frac{1}{2}\right)^{2}m\right). \end{aligned}}  $$

Clearly, for every *n*, every *h*, and every *λ* that satisfies (5), this expression approaches 1 as *m* approaches infinity. This completes the proof. □

Lemma 1 enables one to estimate the number of gene trees required for successful tree reconstruction: If, for example, $e^{-4\lambda h}-\frac {1}{2}=0.1$, then a simple calculation based on Lemma 1 shows that *m*=990 gene trees are sufficient to reconstruct a phylogeny of *n*=100 taxa perfectly with probability of 99%. This amount of gene trees is not uncommon in contemporary studies.

We conclude this section with a remark about the practical implications of the plurality rule: Inequality (5) gives a rather crude approximation for the upper bound of the values of *λ* that allow accurate species tree reconstruction based on the plurality rule (we shall refer to them as “good” values of *λ*), since there are cases in which the plurality rule may point to the correct quartet topology even when its chances of being satisfied by any specific gene tree are less than 50%. For example, when a gene tree has a 40% probability of inducing the correct quartet topology, and 35 and 25% probability of inducing each one of the other two incorrect topologies, the correct quartet topology is deduced with high probability using the plurality inference rule. Moreover, experience shows that wQMC, the reconstruction algorithm used in this paper, may cope successfully with inconsistent input sets (see [44]). These facts imply that accurate tree reconstruction can be achieved in some instances where the value of *λ* exceeds the bound posed by (5). We will later show that this is indeed the case.

### Simulation results

In this section we describe the results of our simulation procedure (see “The simulation procedure” section). We test how HGT events affect individual gene trees and groups of gene trees, and show how the plurality rule can be used for successful tree reconstruction. Though the plurality rule was already used in [43] and studied in [42], our work supplements those papers because here we focus on a large number of quartets simultaneously and find empirical bounds on the rates of HGT that still enable accurate tree reconstruction. Moreover, to the best of our knowledge, this is the first time in which the reconstruction of such large phylogenies (up to 100 taxa in each tree) with the aid of the plurality rule is attempted and reported.

#### The plurality inference rule as a means of reducing HGT-induced noise

When a given species tree undergoes an HGT process, the Qfit score between the species tree and the resulting gene tree indicates of how many quartets in the species tree are preserved despite HGT events. When calculating the Qfit scores between the simulated gene trees and the originating species trees for all tree sizes and all *λ*’s as above, a decrease in the Qfit score that correlates to an increase in the HGT rate was present at all tree sizes, as to be expected. Moreover, the results produced for trees with *n*≥30 were virtually indistinguishable from one another. Hence, this calculation reveals how HGT events affect the gene trees in a quantitative way. See Additional file 1 for more details.

Next, we studied our ability to utilize the gene trees for successful deduction of the species tree’s quartets. Based on [42], quartets topologies were inferred using the plurality rule (see “Reconstructing the species tree based on gene trees” section), and then the Qfit score between the inferred quartets and the species tree was calculated. Thus, the robustness of the plurality rule as a quartet oracle was tested. We refer to the result of this calculation as “plurality Qfit”.

**Fig. 1:**
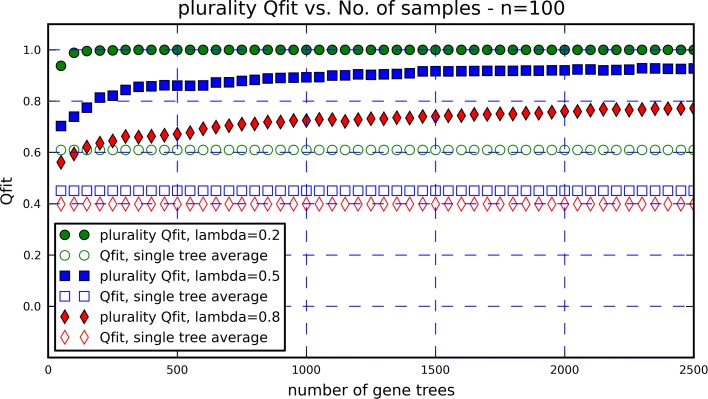
Plurality Qfit and average (single tree) Qfit scores, for three values of *λ* and a constant *n*=100. We note three important features: a) The plurality Qfit may be much greater than the average Qfit. b) The plurality Qfit is an increasing function of the number of gene trees *m*. c) The plurality Qfit is a decreasing function of the HGT rate *λ*

We carried out the aforementioned process for trees of all sizes *n* and all HGT rates *λ* as above. For each fixed *n* and *λ* we generated a graph depicting the plurality Qfit, as a function of the number of gene trees taken under consideration *m*. The graph presented here (Fig. 1, with *n*=100 and *λ*=0.2, 0.5, 0.8) demonstrates the main features of the results. The first noted property is that even though the average Qfit of a gene tree may be quite low, when looking at the plurality Qfit of a large number of gene trees, the fitness score becomes substantially greater. The second feature is that the plurality Qfit score is an increasing function of the number of gene trees. Both these facts imply that the more gene trees are examined, the greater the number of correctly deduced quartet topologies will become. Moreover, these results enable one to estimate how many gene trees one must consider in order to extract the tree-like signal of evolution, and prove that the plurality rule can be used to infer most of the quartet topologies successfully, even at surprisingly high levels of HGT. The third noted feature is that when the number of samples (i.e., gene trees) is constant, the plurality Qfit score is consistently higher for smaller values if *λ*. This means that a high rate of HGT events hinders the efficiency of the plurality inference rule, as is to be expected.

#### Reconstructing the simulated species tree

In this section, the plurality inference rule is used together with wQMC as a tool to reconstruct the simulated species trees. As mentioned in “The simulation procedure” section, ten species trees were created (with *n*=10, 20,…,100 leaves), each species tree accompanied with ten families of gene trees, for *λ*=0.1, 0.2,…,1.0, each family consists of 2500 gene trees. First, a collection of 10000 4-taxa sets was sampled. Next, the plurality quartets of each 4-taxa were inferred based on *m*=250 simulated gene trees and then used as input for wQMC. The process was then repeated for *m*=500, 750,…,2500 gene trees. In order to determine the weighting method best suited for tree reconstruction, wQMC was run with three different weighting methods:

1. The chosen quartets were unweighted (equivalently, were all assigned a weight of 1).
2. The chosen quartets were assigned a weight equal to the number of votes they got. For instance, if the plurality quartet was present in 200 out of 250 gene trees, then its assigned weight was 200.
3. All three topologies of each sampled 4-taxa were added to the input, and the weight of each topology equaled the number of votes it got.

The quality of reconstruction was measured by calculating the Qfit score between the species tree and the reconstructed trees. In order to filter out noises resulting from quartet sampling, the Qfit scores were calculated 10 times. Thus, the data presented in this section is the average Qfit. In general, weighting method No. 2 enabled the most accurate species tree reconstruction. We say that accurate tree reconstruction was reached if the Qfit scores between the species tree and the reconstructed tree (based on method No. 2) were consistently greater than 99% when the number of gene trees sampled was 1500 or more. The *m*=1500 gene trees threshold was set because, experimentally, the Qfit scores were stable when the number of gene trees exceeded 1500, for all HGT rates examined. Obviously, for small values of *λ*, a smaller number of gene trees can suffice. Indeed, when relying on method No. 2, for *λ*≤0.4 and all sizes of species tree, it was enough to sample *m*=500 gene trees in order to achieve accurate tree reconstruction.

In Table 1, the maximum values of *λ* that enabled accurate tree reconstruction in the simulation study, are compared to the maximum values of *λ* that guarantee accurate tree reconstruction according to the known theory (5). In all cases, the empirical values of *λ* that still enable accurate tree reconstruction are much higher (one order of magnitude) than the theoretical-based ones. This fact implies that the plurality rule may be a more powerful tool than what the known theorems suggest. As mentioned earlier (end of “Using the plurality inference rule to reconstruct complete species trees” section), several causes are likely to contribute to this difference between theory and practice. First, using the plurality inference rule, one may deduce the correct quartet topology even if the probability of finding this topology in a given gene tree is less than 50%. Second, experience shows that wQMC may reconstruct the phylogeny accurately even if some quartet topologies are deduced incorrectly, and the plurality quartets are inconsistent [44]. For further discussion about quartet inference in light of HGT events, see Additional file 1.

**Table 1:** Theoretical and empirical values of maximum “good” *λ*’s

Number of leaves	10	20	30	40	50	60	70	80	90	100
Maximum “good” *λ* ^′^*s* - theory	0.07	0.06	0.06	0.04	0.05	0.05	0.04	0.05	0.06	0.04
Maximum “good” *λ* ^′^*s* - practice	1.0	0.6	0.7	0.5	0.6	0.5	0.5	0.4	0.7	0.4

We define maximum “good” *λ* as the maximum value of *λ* that enables us to reconstruct the species tree accurately. Of course, the exact values of the “good” *λ*’s may vary from one species tree to another. As one can see, the empirical values of maximum “good” *λ*’s are much higher than the theoretical ones, dictated by (5) (approximately one order of magnitude)

### Real data analysis

In order to show that the plurality rule can be harnessed for the analysis of real data, we used it (together with wQMC) to construct the hypothesized phylogenies of two sets of organisms. The phylogenies constructed were compared to other hypothesized phylogenies using Qfit, RF and the property of *perfect phylogeny* mentioned above. The first set we studied comprised of 100 archaea and bacteria that cover a wide diversity of the prokaryotic world. This set was first analyzed in depth in [18]. The second set comprised of 97 bacteria, all of which were found to contain genes toxic to *E. coli* [58]. Notice that gene toxicity also implies fewer occurrences of successful HGT events, hence the existence of toxic genes alongside non-toxic genes is expected to result in non-uniform HGT events. It is interesting to examine the usefulness of the plurality rule in such a setting.

#### First set of organisms - wide diversity of prokaryotes

The first set of genes we studied comprised of 100 archaea and bacteria, for which 6901 gene trees were already constructed in [18], and subsequently investigated in [19, 20]. In order to construct a suggested phylogeny for this set of species, we found the plurality quartets based on the available gene trees, and ran wQMC using these quartets as input. All possible $n \choose 4$ quartets were taken into account. Based on the results of the simulation study (“Reconstructing the simulated species tree” section), we used weighting method No. 2. The resulting tree is referred to as *QP1* (quartet plurality based tree 1).

The claim that the plurality rule can be used to reconstruct accurate phylogenies can be reinforced by comparing QP1 to two other suggested trees on the same taxa set. One such suggested phylogeny is found in [18] and was constructed using CLANN based on a subset of *nearly universal trees* (or NUTs) taken strictly from the COG database. We refer to it as the COG tree. The other suggested phylogeny is based on a concatenation of several ribosomal proteins [59]. We refer to it as the ribosomal protein tree. Full details about the phylogenies used in our study are found in Additional file 1.

One of the tools of comparison used was to test whether the trees in question are *perfect* (see “Preliminaries” section) with respect to two characters, induced by two evolutionary classifications - phylum, and order. We briefly mention that a phylogeny is called “perfect” with respect to a given classification if it induces a perfect separation between the different classes of that classification. The results show (Table 2) that two of the three trees examined - the QP1 tree and the ribosomal protein tree - are either perfect or have one misplaced taxon with respect to the two classifications. In addition, the average similarity of the suggested phylogenies to the original input trees was calculated (expressed by Qfit and RF). We see (Table 3), that the QP1 tree and the ribosomal protein tree achieved almost identical results, that are better than the results of the COG tree.

**Table 2:** A summary of the properties of the different characters of the suggested phylogenies

	Classification by phylum	Classification by order
QP1 tree	Tree is not perfect. One leaf misplaced	Tree is perfect
COG tree	Tree is not perfect. Eight leaves misplaced	Tree is not perfect. One leaf misplaced
Ribosomal protein tree	Tree is perfect	Tree is perfect

A tree can either be perfect (i.e., induce a perfect separation with respect to the relevant character) or not, in which case the number of taxa needed to be replaced in order to make the tree perfect is indicated. We see that the ribosomal protein tree is perfect with respect to both characters, and that the QP1 tree is perfect apart from one misplaced taxon

**Table 3:** A summary of the average Qfit and RF similarity scores between the three suggested phylogenies and the underlying gene pool

	Similarity measure	
	Average Qfit	Average RF
QP1 tree	0.48	0.42
COG tree	0.47	0.39
Ribosomal protein tree	0.48	0.42

For each suggested phylogeny, the Qfit and RF similarity scores between it and each gene in the gene pool were calculated. The average similarity scores were subsequently calculated and presented. We see that the scores relating to the QP1 tree and the ribosomal protein tree are the highest

#### Second set of organisms - species with toxic genes

The second set of species we studied comprised of species whose genomes contain genes that are toxic to *E. coli*. In [60] it was found that several genes cannot undergo HGT into *E. coli*. Following that work, several hundreds of such genes were identified [61] and a database named *PanDaTox* containing these toxic genes was constructed [58], from which a representative set of 97 species was selected for further analysis. More details are found in Additional file 1. We stress that evidence was found to suggest the genes listed in PanDaTox are toxic to bacteria in general [60], with yet unknown pattern of HGT, as opposed to the random model assumed in [42]. It is interesting to see how this mixture of toxic and non-toxic genes affect quartet inference and subsequent tree reconstruction based on the plurality rule.

Since four is the minimum number required for the construction of an unrooted tree, the gene set we analyzed comprised of all genes with orthologs in at least four of the above 97 species. Gene orthology was inferred using the EggNOG database [62] (version 3.0). In order to avoid false identification of orthologous genes, we ignored genes that had paralogs in their COG. Thus, only when a species had one representative gene in a COG, was that gene taken under consideration. The exception to that rule was the 16s gene, of which there are typically several copies in each species. We therefore chose one such copy per species (from the RDP website, as mentioned above) and constructed a phylogeny based on the resulting selected set of 16s genes.

In total, 3597 gene clusters were analyzed. The respected gene trees of those gene clusters were constructed using fasttree [52]. We note that a separate hypothesized phylogeny was constructed based solely on the 16s gene using RAxML (see “Reconstructing gene trees based on protein sequences” section). In accordance with the previous section, the plurality inference rule was used to determine which of the three quartet topologies should each 4-taxa be given. Again, all possible $n \choose 4$ quartets were taken under consideration and then used as input for wQMC, with weights assigned according to weighting method No. 2. The resulting tree is denoted QP2.

Again, the quality of reconstruction was evaluated by comparing the tree constructed based on the plurality rule and wQMC, the QP2 tree, to two other suggested phylogenies. The first is the 16s tree mentioned above. The second is a phylogeny based on the concept of *synteny index*, which is a measure of the average synteny between two genomes. As in [63], where this concept was first introduced, we calculated the synteny indices of all possible pairs out of the 97 genomes and constructed a tree based on the resulting distance matrix. We refer to this tree as the synteny tree.

We first checked whether the three phylogenies are perfect or not, with respect to phylum and order. The results show (Table 4) that none of the phylogenies we examined is perfect with respect to order, however the QP2 tree and the 16s tree are perfect with respect to phylum. We subsequently calculated the average similarity scores between the gene trees and each of the three species trees. The results (Table 5) show that the QP2 tree received the highest Qfit and RF scores.

**Table 4:** A summary of the properties of the different characters of the suggested phylogenies

	Classification by phylum	Classification by order
QP2 tree	Tree is perfect	Tree is not perfect. Three leaves misplaced
16s tree	Tree is perfect	Tree is not perfect. Two leaves misplaced
Synteny tree	Tree is not perfect. One leaf misplaced	Tree is not perfect. Three leaves misplaced

A tree can either be perfect (i.e., induce a perfect separation with respect to the relevant character) or not, in which case the number of taxa needed to be replaced in order to make the tree perfect is indicated. We see that no single tree is perfect with respect to order, but the QP2 tree and the 16s tree are perfect with respect to phylum

**Table 5:** A summary of the average Qfit and RF similarity scores between the three suggested phylogenies and the underlying gene pool

	Similarity measure	
	Average Qfit	Average RF
QP2 tree	0.69	0.47
16s tree	0.66	0.39
Synteny tree	0.67	0.42

For each suggested phylogeny, the Qfit and RF similarity scores between it and each gene in the gene pool were calculated. The average similarity scores were subsequently calculated and presented. We see that the QP2 tree receives the highest average similarity scores

## Discussion

This study focuses on phylogenetic reconstruction in light of extensive HGT events, based on the quartet plurality inference rule. As mentioned earlier, HGT tangles the universal Tree of Life, turning it into a network of relationships. In the context of HGT, phylogenetic reconstruction often involves gene tree amalgamation (the supertree approach). Quartets are of prime importance for the supertree approach, as they are the most fundamental unit of information when unrooted trees are studies. A thorough investigation of the plurality inference rule enabled us to expand a past theoretical result [42], as well as to show using real and simulated data, that accurate quartet-based phylogenetic reconstruction can be reached.

The results of our investigation give rise to some questions for future research. From a theoretical perspective, in light of our capability of reconstructing accurate evolutionary trees despite surprisingly high rates of HGT, we hypothesize that an improvement can be made to the known upper bound on the HGT rate *λ* that still enables successful tree reconstruction (given in (5)). Moreover, our conjecture is that in our theoretical framework, correct quartet inference can be achieved for *all* HGT rates, provided that the number of available gene trees is sufficiently large. We base our conjecture on the (yet unsubstantiated) claim that each one of the two incorrect topologies of a quartet has equal probability to be induced by a given species tree. If that claim is proven, we believe that the positive probability of zero HGT events will tip the balance in favour of the correct quartet topology. Regarding phylogenetic reconstruction based on real data, we mention that we considered merely three weighting schemes and used one supertree method, wQMC. Naturally, future studies may reveal that other supertree methods are better equipped to deal with inputs comprised of quartet trees. Moreover, since assigning weight to a quartet can be done in any number of ways, we think some attention should be allocated for searching new weighting schemes, perhaps based on the evolutionary distances between the different species under study, that will improve the accuracy of tree reconstruction. We will welcome any result that will improve our own.

## Conclusions

In this work we investigated the plurality inference rule using real and simulated data. First, by considering a multitude of quartets simultaneously, we were able to evaluate the number of gene trees needed to facilitate accurate species tree reconstruction with high probability, thus we added to the theoretical analysis of [42]. Subsequently, through an in-depth simulative study, we proved that accurate species tree reconstruction is possible in the presence of extensive HGT events. In the last segment of our work, we constructed hypothesized phylogenies of two sets of prokaryotic species, one encompassing a wide diversity of the prokaryotic world and the other comprised solely of species with toxic genes, and showed that our hypothesized phylogenies achieved similar results to other suggested evolutionary trees in a number of tests. To the best of our knowledge, this is the first time that the quartet plurality inference rule was used to construct real data phylogenies of such magnitude.

## Additional file

Additional file 1 A supplementary text to the main body of the paper. (PDF 1587 kb)
