# Supplementary material for: Reconstruction of real and simulated phylogenies based on quartet plurality inference
Source: BMC Genomics. 2018 Aug 13;19(Suppl 6):570. doi: 10.1186/s12864-018-4921-5 (PMC6101080; doi:10.1186/s12864-018-4921-5)
Supplement: Supplementary file 1 — A supplementary text to the main body of the paper. (PDF 1587 kb) [file 12864_2018_4921_MOESM1_ESM.pdf]

APPENDIX A: SUPPLEMENTARY TEXT

This is a supplementary text to "Reconstruction of Real and Simulated Phylogenies Based on Quartet Plurality Inference". We start by elaborating on the simulation procedure that was used in the paper, then state and prove a lemma concerning the probability of correct reconstruction of all quartets induced by a species tree. In the following section we present the average Qfit score between species trees and gene trees of varying sizes, as a function of the HGT rate λ. This section is followed by a discussion about the probability of a given quartet to be altered by HGT events, depending on several of its parameters. We conclude this supplementary text in a short segment in which the details of the real data used in the paper are given.

1. The simulation procedure

We produced ten random, edge-weighted, simulated species trees over n taxa (for $n = 10, 20, \dots 100$). Each simulated species tree was produced based on the Yule process (see [5]). Subsequently, we generated ten families of "gene" trees for each simulated species tree, each family consisting of 2500 gene trees. Each gene tree simulates a tree that is created when a species tree is subjected to a certain HGT process - a series of HGT events, consistent with a Poisson process of a constant rate. Ten different HGT rates were used, namely $\lambda = 0.1, 0.2, \dots, 1.0$. The rate of the HGT process remained constant within each gene family, but varied from one gene family to another. The simulated species trees and gene trees were created using our own scripts, incorporated in the supplementary material. The details of how the model trees and the gene trees were created appear below.

1.1. Generating a simulated species tree.

Our simulated species trees were generated based on the Yule procees. The Yule process is a process in which a binary ultrametric tree is generated. Generating a Yule tree with n leaves is a process that has $n-1$ recursive stages, where in a preliminary stage, a node that represents the root of the tree is created with a time signature of zero.

In the first stage of the recursion, two exponentially distributed numbers are sampled. Let us denote them as T_1 and T_2. We choose the exponential distribution to have a parameter $\lambda = 1$, which means that for every $\varepsilon > 0$ we have $P(T_1 < \varepsilon) = P(T_2 < \varepsilon) = 1 - e^{-\varepsilon}$. After T_1 and T_2 are set, two nodes are created with time signatures T_1 and T_2. They are then connected to the root as two descendant leaves. (We can think of the time signature as representing the time of that node's creation. Thus, nodes with small time signatures are considered as "created" before nodes with large ones.) This completes the first stage. Note that once this stage is completed, there are two leaves in our tree.

Let us assume that the $(k-1)$-th stage in the recursion has been completed, resultin in a tree with k leaves. We carry out the k-th stage as follows: Two exponentially distributed numbers are sampled, let us denote them as $T_1^{(k)}$ and $T_2^{(k)}$, and two nodes are created. These two nodes are connected as descendant leaves to the leaf of the $k-1$ stage that has the smallest time signature (the leaf that was "created" first, which now becomes an inner node of the tree), and their time signatures are set at $T_1^{(k)}$ (or $T_2^{(k)}$) *plus* the time signature of their immediate ancestor. (Naturally, the time signature of each node on the Yule tree may be regarded as representing its distance from the root, or the *length* of the path from it to the root.) It is easy to see that at this point the generated tree has $k+1$ leaves. This completes the k-th stage in the recursive process.

Once $n-1$ stages have been completed and we have generarted a tree with n leaves, the time signatures of all the leaves are reset to equal the smallest time signature among the leaves. This final act is what makes the generated tree ultremetric.

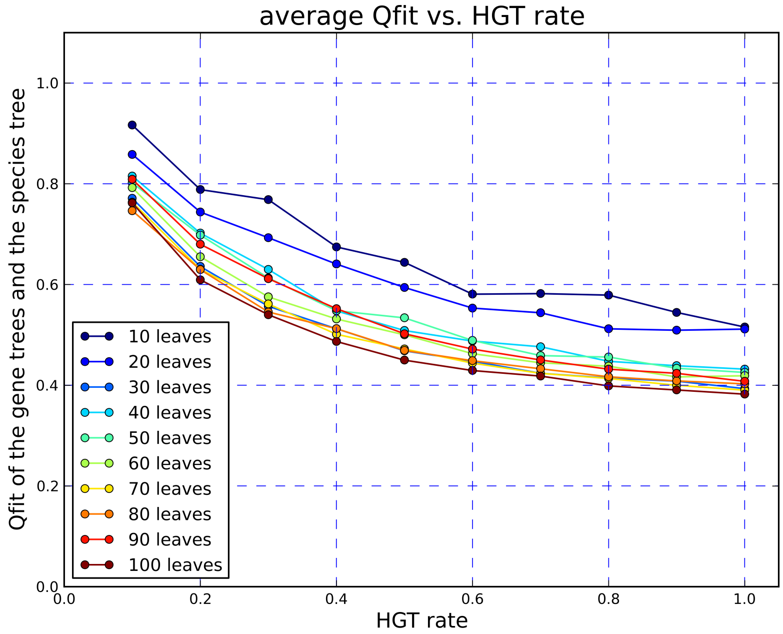

FIGURE 1. The Qfit score of the gene trees and the species tree, as a function of the rate of HGT λ. The scores are averaged over 100 runs. We see that the average Qfit score is a decreasing function of the HGT rate.

1.2. **Generating a simulated gene tree.** Based on a given species tree, we generated a set of *gene trees* by subjecting the species tree to a Poisson process of HGTs with a constant rate of events λ. This means that for each gene tree, the number of HGT events, denoted by $\#LGT$, was a random variable for which $Pr(\#LGT = m) = \frac{e^{-\lambda L}(\lambda L)^m}{m!}$ (where L denotes the total length of the species tree). Furthermore, once the value of $\#LGT$ was fixed, the m recipients of HGT events were distributed randomly and uniformly on the species tree. We remark that this is equivalent to assuming that the time between two HGT events along each lineage is an exponentially distributted random varialbe (like speciation events in the Yule model). An HGT event is simulated by a subtree pruning and regrafting (SPR) operation (see [3]), from the recipient of genetic material to another, randomly chosen point, at the same depth from the root in the species tree (i.e., a contemporaneous species) - the donor. When all HGTs for a gene were generated, the resulted gene tree was ready.

2. Qfit between the gene trees and the species tree

A natural question to ask, once we subject a given species tree to an HGT process, is how the latter affects the similarity between that species tree and the new gene tree. This similarity, as expressed by the Qfit score, is an indication of how many quartets in the species tree remain unchanged after being subjected to HGT events. To answer this question, we plotted the average Qfit score between the simulated gene trees and the originating species trees for all tree sizes and all λ as above. As expected, a decrease in the Qfit score that correlates to an increase in the HGT rate, was present at all tree sizes. Moreover, the results produced for trees with $n \geq 30$ were virtually indistinguishable from one another (see Figure 1). Hence, this calculation reveals how HGT events affect the gene trees in a quantitative way.

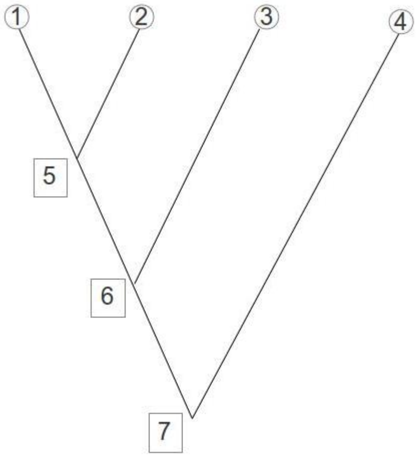

FIGURE 2. An example of a rooted quartet. The distance between the two closest leaves, in this case the $(1, 2)$-cherry, is the most important feature influencing the quartet's chances of being altered by HGT events.

3. Further discussion about quartet inference: Which quartets are most affected by HGT?

In this part we establish a connection between the quartet's structure, as it appears in the species tree, and the quartet's chances of being altered by HGT. For two leaves x, y on a tree, we denote the distance between them as $d_{x,y}$. As can be illustrated by Figure 2, which shows an example of a rooted quartet, any quartet has several parameters induced by it. We chose to focus on: The "long" sum (in our case, $d_{1,3} + d_{2,4} = d_{1,4} + d_{2,3}$), the "short" sum (in our case, $d_{1,2} + d_{3,4}$), the length of the middle branch ($d_{5,6}$), the distance between the two closest leaves ($d_{1,2}$), and the quartet's weight, as defined in [1] $(1 - \frac{d_{1,2}+d_{3,4}}{d_{1,3}+d_{2,4}})$.

As in the main body of the text, we define "success" of a quartet as the event in which the topology of a given 4-taxa, as it is induced by a gene tree, is identical to the topology of the same 4-taxa as it is induced by the species tree. We refer to the "success rate" of a quartet as the probability of the event "success". To find a connection between the success rate and one of the quartet's parameters, we carried out the following process: We sampled a set of 10000 quartets randomly. While taking a collection of 1000 gene trees under consideration, we counted the number of gene trees in which the topology of each quartet was equal to the original topology induced by the model tree. We then normalized the results by dividing that number by the total number of gene trees (1000). This gives us an estimation of the success rate of each quartet. We then plotted the results in a two dimensional graph, where each point of the graph represents a quartet, and its (x, y) coordinates correspond to the parameters we examined: x - the "long" sum / "short" sum / quartet weight etc. (depending on context), y - the success rate. Graphs were generated for species trees of all sizes and HGT rates as above. Figure 3 that we present here offers a good representative example of the results we got.

Among the different parameters that we examined, we concluded that the distance between the two closest leaves has the most profound effect on the success rate of a quartet, especially when the rate of HGT events (reflected in a large value of λ) is high. We see (Figure 3) that the "long" sum is uncorrelated with the success rate of a quartet. As for the the "short" sum, the length of the middle branch, and the quartet's weight: there is some correlation between them and the success rate. However, an almost perfect one to one correspondence between the variables is only present when looking at the correlation between the distance of the closest leaves and the success rate. This is due to the fact that the distance between the two closest leaves is, by definition, exactly twice the

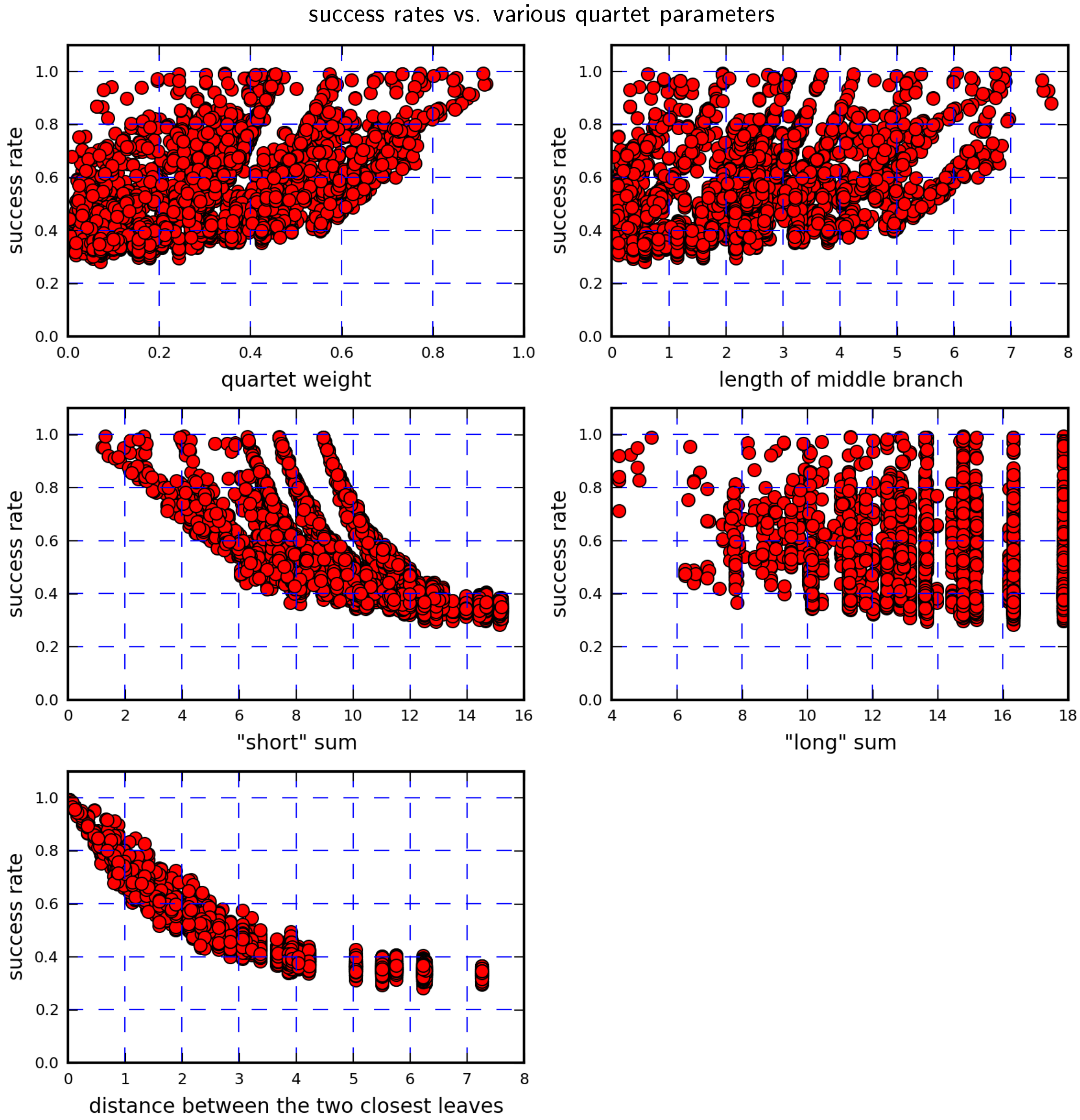

FIGURE 3. The success rate of a species tree quartets with respect to several of the quartets' parameters. As can be seen, there is a strong one-to-one correlation between the distance between the two closest leaves and the success rate of the quartet (lower left plot). $n = 100$, $\lambda = 0.5$.

distance between each of those leaves and their last common ancestor (see leaves 1,2 and node 5 in Figure 2). It is easy to see that any HGT event occurring prior to the time of that latest bifurcation (before node 5 in our example) would have no chance of changing the quartet's topology, as it cannot "break" the 1,2 cherry.

4. Full details about the real data used in our study

In the main body of the text we analyzed two sets of prokaryotic speices. Here we provide the details of those two sets and their respected phylogenies.

The first set of prokaryotes: A full list of the first set of prokaryotes is given in Tables 1,2. This list can also be found in [2], where it was first investigated. In our paper, we considered three phylogenies relating to this set, denoted QP1 tree, COG tree, and ribosomal protein tree. The QP1 tree was constructed by ourselves, the COG tree was constructed in [2] where it was published as a figure (and later given to us via private communication), and the ribosomal protein tree is found in supplementary file S1 of [4] (the ST_3 tree of that paper). All three trees are given, in Newick format, in Table 3.

The second set of prokaryotes: A full list of the second set of prokaryotes is given in Tables 4,5,6. In our paper, we considered three phylogenies relating to this sets, denoted QP2 tree, 16s tree, and synteny tree. All three phylogenies were constructed by ourselves, as is described in the main body of the text. They are given, in Newick format, in Table 7.

Table 1. The full list of species belonging to the first set of prokaryotes of our study, part 1 of 2.

number	full name	phylum	order
0	Picrophilus torridus DSM 9790	Euryarchaeota	Thermoplasmales
1	Thermoplasma volcanium	Euryarchaeota	Thermoplasmales
2	Thermoplasma acidophilum	Euryarchaeota	Thermoplasmales
3	Cenarchaeum symbiosum	Crenarchaeota	Cenarchaeales
4	Methanopyrus kandleri	Euryarchaeota	Methanopyrales
5	Methanobacterium thermoautotrophicum	Euryarchaeota	Methanobacteriales
6	Methanosphaera stadtmanae	Euryarchaeota	Methanobacteriales
7	Methanococcus jannaschii	Euryarchaeota	Methanococcales
8	Methanococcus maripaludis S2	Euryarchaeota	Methanococcales
9	Methanococcus maripaludis C5	Euryarchaeota	Methanococcales
10	Lentisphaera araneosa HTCC2155	Lentisphaerae	Lentisphaerales
11	Clostridium acetobutylicum ATCC 824	Firmicutes	Clostridiales
12	Cytophaga hutchinsonii ATCC 33406	Bacteroidetes	Sphingobacteriales
13	Flavobacterium johnsoniae UW101	Bacteroidetes	Flavobacteriales
14	Chlorobium tepidum TLS	Chlorobi	Chlorobiales
15	Prosthecochloris vibrioformis DSM 265	Chlorobi	Chlorobiales
16	Desulfovibrio vulgaris subsp vulgaris str Hildenborough	Proteobacteria-Delta	Desulfovibrionales
17	Aquifex aeolicus VF5	Aquificae	Aquificales
18	Bacillus subtilis subsp subtilis str 168	Firmicutes	Bacillales
19	Rubrobacter xylanophilus DSM 9941	Actinobacteria	Rubrobacterales
20	Chloroflexus aurantiacus J-10-fl	Chloroflexi	Chloroflexales
21	Gloeobacter violaceus PCC 7421	Cyanobacteria	Gloeobacterales
22	Prochlorococcus marinus subsp marinus str CCMP1375	Cyanobacteria	Prochlorales
23	Anabaena variabilis ATCC 29413	Cyanobacteria	Nostocales
24	Nostoc sp PCC 7120	Cyanobacteria	Nostocales
25	Thermosynechococcus elongatus BP-1	Cyanobacteria	Chroococcales
26	Acaryochloris marina MBIC11017	Cyanobacteria	unclassified
27	Trichodesmium erythraeum IMS101	Cyanobacteria	Oscillatoriales
28	Synechocystis sp PCC 6803	Cyanobacteria	Chroococcales
29	Moorella thermoacetica ATCC 39073	Firmicutes	Thermoanaerobacteriales
30	Acidobacteria bacterium Ellin345	Acidobacteria	Acidobacteriales
31	Myxococcus xanthus DK 1622	Proteobacteria-Delta	Myxococcales
32	Solibacter usitatus Ellin6076	Acidobacteria	Solibacterales
33	Mycobacterium tuberculosis H37Rv	Actinobacteria	Actinomycetales
34	Blastopirellula marina DSM 3645	Planctomycetes	Planctomycetales

Table 2. The full list of species belonging to the first set of prokaryotes of our study, part 2 of 2.

number	full name	phylum	order
35	Rhodopirellula baltica SH 1	Planctomycetes	Planctomycetales
36	Gemmata obscuriglobus	Planctomycetes	Planctomycetales
37	Planctomyces maris DSM 8797	Planctomycetes	Planctomycetales
38	Deinococcus radiodurans R1	Deinococci	Deinococcales
39	Thermus thermophilus HB27	Deinococci	Thermales
40	Helicobacter pylori 26695	Proteobacteria-Epsilon	Campylobacterales
41	Sulfurovum sp NBC37-1	Proteobacteria-Epsilon	unclassified
42	Escherichia coli K12	Proteobacteria-Gamma	Enterobacteriales
43	Methylococcus capsulatus str Bath	Proteobacteria-Gamma	Methylococcales
44	Pseudomonas aeruginosa PAO1	Proteobacteria-Gamma	Pseudomonadales
45	Burkholderia mallei ATCC 23344	Proteobacteria-Beta	Burkholderiales
46	Neisseria meningitidis MC58	Proteobacteria-Beta	Neisseriales
47	Methylibium petroleiphilum PM1	Proteobacteria-Beta	Burkholderiales
48	Methylobacillus flagellatus KT	Proteobacteria-Beta	Methylophilales
49	Chlamydia trachomatis D/UW-3/CX	Chlamydiae	Chlamydiales
50	Chlamydophila pneumoniae AR39	Chlamydiae	Chlamydiales
51	Candidatus Protochlamydia amoebophila UWE25	Chlamydiae	Chlamydiales
52	Methylokorus infernorum V4	Verrucomicrobia	Verrucomicrobiales
53	Leptospira interrogans serovar Copenhageni str Fiocruz L1-130	Spirochaetes	Spirochaetales
54	Fusobacterium nucleatum subsp nucleatum ATCC 25586	Fusobacteria	Fusobacteriales
55	Methanoculleus marisnigri JR1	Euryarchaeota	Methanomicrobiales
56	Methanospirillum hungatei JF-1	Euryarchaeota	Methanomicrobiales
57	Methanocorpusculum labreanum Z	Euryarchaeota	Methanomicrobiales
58	Uncultured methanogenic archaeon	Euryarchaeota	?
59	Archaeoglobus fulgidus	Euryarchaeota	Archaeoglobales
60	Methanococcoides burtonii DSM 6242	Euryarchaeota	Methanosarcinales
61	Methanosarcina barkeri fusaro	Euryarchaeota	Methanosarcinales
62	Methanosarcina acetivorans	Euryarchaeota	Methanosarcinales
63	Methanosarcina mazei	Euryarchaeota	Methanosarcinales
64	Methanosaeta thermophila PT	Euryarchaeota	Methanosarcinales
65	Caldivirga maquilingensis IC-167	Crenarchaeota	Thermoproteales
66	Thermoproteus tenax	Crenarchaeota	Thermoproteales
67	Pyrobaculum islandicum DSM 4184	Crenarchaeota	Thermoproteales
68	Pyrobaculum calidifontis JCM 11548	Crenarchaeota	Thermoproteales
69	Pyrobaculum aerophilum	Crenarchaeota	Thermoproteales
70	Sulfolobus solfataricus	Crenarchaeota	Sulfolobales
71	Sulfolobus acidocaldarius DSM 639	Crenarchaeota	Sulfolobales
72	Sulfolobus tokodaii	Crenarchaeota	Sulfolobales
73	Aeropyrum pernix	Crenarchaeota	Desulfurococcales
74	Hyperthermus butylicus	Crenarchaeota	Desulfurococcales
75	Natronomonas pharaonis	Euryarchaeota	Halobacteriales
76	Halobacterium sp	Euryarchaeota	Halobacteriales
77	Haloquadratum walsbyi	Euryarchaeota	Halobacteriales
78	Haloarcula marismortui ATCC 43049	Euryarchaeota	Halobacteriales
79	Opitutaceae bacterium TAV2	Verrucomicrobia	Opitutales
80	Agrobacterium tumefaciens str C58	Proteobacteria-Alpha	Rhizobiales
81	Bacteroides thetaiotaomicron VPI-5482	Bacteroidetes	Bacteroidales
82	Pyrococcus horikoshii	Euryarchaeota	Thermococcales
83	Pyrococcus abyssi	Euryarchaeota	Thermococcales
84	Pyrococcus furiosus	Euryarchaeota	Thermococcales
85	Thermococcus kodakaraensis KOD1	Euryarchaeota	Thermococcales
86	Victivallis vadensis ATCC BAA-548	Lentisphaerae	Victivallales
87	Thermotoga maritima MSB8	Thermotogae	Thermotogales
88	Dehalococcoides sp BAV1	Chloroflexi	Dehalococcoidetes
89	Bifidobacterium longum NCC2705	Actinobacteria	Bifidobacteriales
90	Methylobacterium extorquens PA1	Proteobacteria-Alpha	Rhizobiales
91	Staphylothermus marinus F1	Crenarchaeota	Desulfurococcales
92	Thermofilum pendens Hrk 5	Crenarchaeota	Thermoproteales
93	Lactobacillus casei ATCC 334	Firmicutes	Lactobacillales
94	Fervidobacterium nodosum Rt17-B1	Thermotogae	Thermotogales
95	Mesoplasma florum L1	Firmicutes	Entomoplasmatales
96	Borrelia burgdorferi B31	Spirochaetes	Spirochaetales
97	Treponema pallidum subsp pallidum str Nichols	Spirochaetes	Spirochaetales
98	Rickettsia prowazekii str Madrid E	Proteobacteria-Alpha	Rickettsiales
99	Nanoarchaeum equitans	Nanoarchaeota	?

Table 3. The three phylogenies constructed based on the first set of prokaryotes (in Newick format).

Tree name	Tree string
QP1 tree	((((((((((62,63),61),60),64),58),((55,56),57)),(((75,78),76),77)),59),((((8,9),7),(5,6)),4)),(((((((((67,69),68),66),65),92),(((71,72),70),((73,74),91))),99),3),(((82,83),84),85)),((1,2),0))),(((((((((((((45,47),48),46),((42,44),43)),((80,90),98)),16),(40,41)),((30,32),31)),(((((34,35),37),36),(((49,50),51),((10,86),(52,79)))),((((12,13),81),(14,15)),53))),(96,97)),(((((((((23,24),27),26),28),25),22),21),20),(((33,89),19),(38,39))),((((18,93),11),29),88))),95),54),((87,94),17)));
COG tree	((((((((((49,50),51),96),((10,79),97)),((((34,35),37),36),(86,52))),(((((81,13),12),(15,14)),(39,11)),((30,32),53))),(((((((((23,24),27),28),(26,25)),22),21),29),(87,19)),((((18,93),95),(17,94)),((88,33),(89,20))))),(((((((45,47),48),46),((44,42),43)),((80,90),98)),16),(((41,40),54),31))),38),((((((((((78,76),(77,75)),(55,56)),(((((62,63),61),60),57),59)),4),((((8,9),7),(6,5)),((1,2),0))),(((((((69,67),68),66),65),92),((((72,71),70),73),(74,91))),((((82,83),84),85),3))),58),64),99));
Ribosomal protein tree	(((3,((92,(65,(66,(68,(69,67))))),((73,(91,74)),(70,(72,71))))),(99,((85,(84,(83,82))),(4,(((5,6),(7,(8,9))),((0,(1,2)),(59,((76,(77,(75,78))),((57,(56,55)),(58,(64,(60,(61,(62,63))))))))))))))),((((53,(97,96)),(((14,15),(12,(81,13))),((36,(37,(34,35))),(((86,10),(52,79)),(51,(50,49)))))),((((39,38),(19,(89,33))),((88,20),(21,(22,((26,25),(28,(27,(24,23)))))))),((17,(94,87)),((54,95),((29,11),(18,93)))))),((32,30),((41,40),((31,16),((98,(90,80)),((46,(48,(47,45))),(43,(44,42)))))))));

Table 4. The full list of species belonging to the second set of prokaryotes of our study, part 1 of 3.

Serial number	Full name	phylum	order
0	Bacillus cereus ATCC 10987	Firmicutes	Bacillales
1	Bacillus anthracis str. 'Ames Ancestor'	Firmicutes	Bacillales
2	Bacillus anthracis str. Sterne	Firmicutes	Bacillales
3	Bacillus thuringiensis serovar konkukian str. 97-27	Firmicutes	Bacillales
4	Bacillus cereus E33L	Firmicutes	Bacillales
5	Campylobacter curvus 525.92	Proteobacteria	Campylobacterales
6	Hydrogenobaculum sp. Y04AAS1	Aquificae	Aquificales
7	Bacillus cereus AH187	Firmicutes	Bacillales
8	Bacillus cereus B4264	Firmicutes	Bacillales
9	Geobacillus sp. WCH70	Firmicutes	Bacillales
10	Desulfotomaculum acetoxidans DSM 771	Firmicutes	Clostridiales
11	Thiomicrospira crunogena XCL-2	Proteobacteria	Thiotrichales
12	Methanococcoides burtonii DSM 6242	Euryarchaeota	Methanosarcinales
13	Leuconostoc mesenteroides subsp. mesenteroides ATCC 8293	Firmicutes	Lactobacillales
14	Vibrio cholerae O395	Proteobacteria	Vibrionales
15	Treponema denticola ATCC 35405	Spirochaetes	Spirochaetales
16	Psychrobacter cryohalolentis K5	Proteobacteria	Pseudomonadales
17	Lactobacillus gasseri ATCC 33323	Firmicutes	Lactobacillales
18	Streptococcus thermophilus LMD-9	Firmicutes	Lactobacillales
19	Desulfotomaculum reducens MI-1	Firmicutes	Clostridiales

TABLE 5. The full list of species belonging to the second set of prokaryotes of our study, part 2 of 3.

Serial number	Full name	phylum	order
21	Thermotoga petrophila RKU-1	Thermotogae	Thermotogales
22	Lactobacillus reuteri DSM 20016	Firmicutes	Lactobacillales
23	Staphylococcus aureus subsp. aureus JH1	Firmicutes	Bacillales
24	Marinomonas sp. MWYL1	Proteobacteria	Oceanospirillales
25	Shewanella pealeana ATCC 700345	Proteobacteria	Alteromonadales
26	Desulfitobacterium hafniense DCB-2	Firmicutes	Clostridiales
27	Colwellia psychrerythraea 34H	Proteobacteria	Alteromonadales
28	Chromohalobacter salexigens DSM 3043	Proteobacteria	Oceanospirillales
29	Shewanella sp. MR-7	Proteobacteria	Alteromonadales
30	Pelobacter propionicus DSM 2379	Proteobacteria	Desulfuromonadales
31	Clostridium thermocellum ATCC 27405	Firmicutes	Clostridiales
32	Shewanella putrefaciens CN-32	Proteobacteria	Alteromonadales
33	Yersinia pseudotuberculosis IP 31758	Proteobacteria	Enterobacteriales
34	Shewanella woodyi ATCC 51908	Proteobacteria	Alteromonadales
35	Prosthecochloris aestuarii DSM 271	Chlorobi	Chlorobiales
36	Geobacter bemidjiensis Bem	Proteobacteria	Desulfuromonadales
37	Aliivibrio salmonicida LFI1238	Proteobacteria	Vibrionales
38	Pectobacterium carotovorum subsp. carotovorum PC1	Proteobacteria	Enterobacteriales
39	Moorella thermoacetica ATCC 39073	Firmicutes	Thermoanaerobacterales
40	Desulfomicrobium baculatum DSM 4028	Proteobacteria	Desulfovibrionales
41	Dehalococcoides ethenogenes 195	Chloroflexi	Dehalococcoidales
42	Pseudomonas syringae pv. syringae B728a	Proteobacteria	Pseudomonadales
43	Geobacter metallireducens GS-15	Proteobacteria	Desulfuromonadales
44	Nitrosospira multiformis ATCC 25196	Proteobacteria	Nitrosomonadales
45	Methylobacillus flagellatus KT	Proteobacteria	Methylophilales
46	Shewanella denitrificans OS217	Proteobacteria	Alteromonadales
47	Pseudoalteromonas atlantica T6c	Proteobacteria	Alteromonadales
48	Shewanella frigidimarina NCIMB 400	Proteobacteria	Alteromonadales
49	Shewanella sp. ANA-3	Proteobacteria	Alteromonadales
50	Shewanella amazonensis SB2B	Proteobacteria	Alteromonadales
51	Shewanella baltica OS185	Proteobacteria	Alteromonadales
52	Escherichia coli HS	Proteobacteria	Enterobacteriales
53	Campylobacter concisus 13826	Proteobacteria	Campylobacterales
54	Salmonella enterica subsp. enterica serovar Agona str. SL483	Proteobacteria	Enterobacteriales
55	Acidithiobacillus ferrooxidans ATCC 53993	Proteobacteria	Acidithiobacillales
56	Salmonella enterica subsp. enterica serovar Dublin str. CT_02021853	Proteobacteria	Enterobacteriales
57	Shewanella baltica OS223	Proteobacteria	Alteromonadales
58	Acidithiobacillus ferrooxidans ATCC 23270	Proteobacteria	Acidithiobacillales
59	Dickeya dadantii Ech703	Proteobacteria	Enterobacteriales
60	Slackia heliotrinireducens DSM 20476	Actinobacteria	Coriobacteriales
61	Pelobacter carbinolicus DSM 2380	Proteobacteria	Desulfuromonadales
62	Shewanella sp. MR-4	Proteobacteria	Alteromonadales
63	Enterobacter sp. 638	Proteobacteria	Enterobacteriales
64	Roseiflexus castenholzii DSM 13941	Chloroflexi	Chloroflexales
65	Anaerocellum thermophilum DSM 6725	Firmicutes	Thermoanaerobacterales
66	Methylococcus capsulatus str. Bath	Proteobacteria	Methylococcales
67	Bacillus cereus G9842	Firmicutes	Bacillales
68	Syntrophobacter fumaroxidans MPOB	Proteobacteria	Syntrophobacterales
69	Halothermothrix orenii H 168	Firmicutes	Halanaerobiales
70	Eggerthella lenta DSM 2243	Actinobacteria	Coriobacteriales
71	Jonesia denitrificans DSM 20603	Actinobacteria	Actinomycetales
72	Anaerococcus prevotii DSM 20548	Firmicutes	Clostridiales
73	Wolbachia endosymbiont of Drosophila melanogaster	Proteobacteria	Rickettsiales
74	Clostridium perfringens SM101	Firmicutes	Clostridiales
75	Clostridium perfringens ATCC 13124	Firmicutes	Clostridiales
76	Pelodictyon phaeoclathratiforme BU-1	Chlorobi	Chlorobiales
77	Escherichia coli O157:H7 str. EC4115	Proteobacteria	Enterobacteriales
78	Atopobium parvulum DSM 20469	Actinobacteria	Coriobacteriales
79	Chlorobium luteolum DSM 273	Chlorobi	Chlorobiales
80	Magnetococcus sp. MC-1	Proteobacteria	Magnetococcales
81	Elusimicrobium minutum Pei191	Elusimicrobia	Elusimicrobiales
82	Chlorobium phaeobacteroides BS1	Chlorobi	Chlorobiales
83	Chitinophaga pinensis DSM 2588	Bacteroidetes	Sphingobacteriales
84	Saccharophagus degradans 2-40	Proteobacteria	Alteromonadales

Table 6. The full list of species belonging to the second set of prokaryotes of our study, part 3 of 3.

Serial number	Full name	phylum	order
85	Maricaulis maris MCS10	Proteobacteria	Rhodobacterales
86	Dyadobacter fermentans DSM 18053	Bacteroidetes	Cytophagales
87	Desulfococcus oleovorans Hxd3	Proteobacteria	Desulfobacterales
88	Dickeya zeae Ech1591	Proteobacteria	Enterobacteriales
89	Burkholderia ambifaria AMMD	Proteobacteria	Burkholderiales
90	Streptococcus agalactiae 2603V/R	Firmicutes	Lactobacillales
91	Chlorobium limicola DSM 245	Chlorobi	Chlorobiales
92	Cryptobacterium curtum DSM 15641	Actinobacteria	Coriobacteriales
93	Chlorobium phaeobacteroides DSM 266	Chlorobi	Chlorobiales
94	Candidatus Desulforudis audaxviator MP104C	Firmicutes	Clostridiales
95	Pedobacter heparinus DSM 2366	Bacteroidetes	Sphingobacteriales
96	Yersinia pestis Angola	Proteobacteria	Enterobacteriales
97	Desulfovibrio desulfuricans subsp. desulfuricans str. ATCC 27774	Proteobacteria	Desulfovibrionales

Table 7. The three phylogenies constructed based on the second set of prokaryotes (in Newick format).

Tree name	Tree string
QP2 tree	((((((((((((((((51,57),32),(49,62,29)),(48,46)),(34,25)),50),27),(((((54,56),(77,52)),63),((33,96),(38,88,59))),(14,37))),47),((84,42,24),28)),16),11),66),((44,45),89)),(58,55)),(((((((((((((((((2,1),3),4),(0,7)),(67,8)),9),23),(((22,13),17),(90,18))),(((75,74),72),(65,31))),(((((19,10),94),39),26),69)),((20,41),64)),(((78,(92,70)),60),71)),(21,6)),(((((93,91,76),79),(82,35)),((83,95),86)),(15,81))),(5,53)),((((36,43,30),61),(87,68)),(97,40))),((73,85),80)));
16s tree	(((70,(92,60)),78),(((((((5,53),((80,(85,73)),((55,58),((89,(44,45)),(((16,28),((((((11,47),27),(((((38,(63,(54,((52,77),56)))),(88,59)),(96,33)),(37,14)),((34,25),(((62,29),49),(((48,46),32),(50,(57,51))))))),84),42),24)),66))))),((87,((61,(36,(43,30))),68)),(97,40))),(15,((79,(91,((93,76),(35,82)))),(83,(86,95))))),(((72,(75,74)),31),((((39,94),(26,(10,19))),69),(((((90,18),((17,22),13)),23),(7,(1,((0,(4,3)),((8,67),2))))),9)))),65),(81,((64,(20,41)),(21,6)))),71);
Synteny tree	(((((((((75,74),((69,((((10,19),94),39),26)),((((((2,(3,4)),1),(8,7)),67),0),9),23))),72),(31,65)),((((70,92),60),78),71)),(((22,17),13),(18,90))),21),(41,20)),(((((((((((((((77,52),63),(56,54)),((38,(59,88)),(96,33))),(37,14)),((46,((((((49,(29,62)),(57,51)),32),50),48),(34,25))),(27,47))),((84,(28,42)),24)),11),((45,44),(58,55))),89),66),16),(((((36,43),61),30),(80,85)),((40,97),(87,68)))),15),((((5,53),((76,((35,82),((91,79),93))),((95,83),86))),(6,73)),64)),81);

References

[1] E. Avni, R. Cohen, and S. Snir. Weighted quartets phylogenetics. *Syst Biol*, 64(2):233–242, 2015.

[2] Pere Puigbó, Yuri Wolf, and Eugene Koonin. Search for a 'tree of life' in the thicket of the phylogenetic forest. *Journal of Biology*, 8(6):59, 2009.

[3] C. Semple and M.A. Steel. *Phylogenetics*. Oxford University Press, 2003.

[4] S. Snir, Y. Wolf, and E. Koonin. Universal pacemaker of genome evolution. *PLoS Comput Biol*, 8(11), 11 2012.

[5] G.U. Yule. A mathematical theory of evolution based on the conclusions of Dr. J. C. Willis, F. R. S.. *Philos Trans R Soc Lond B Biol Sci*, 213:21–87, 1924.
